# *FLCN* c.1300G>A: Selective advantage in medieval France

**DOI:** 10.1016/j.gendis.2025.102018

**Published:** 2025-12-30

**Authors:** Agathe Hercent, Ibrahima Ba, Dimitri Tchernitchko

**Affiliations:** Department of Genetics, Bichat Hospital (Assistance Publique Hopitaux de Paris), Reference Laboratory for the Diagnosis of Birt Hogg Dube Syndrome, University of Paris Cité, 46 Rue Henri Huchard, 75018 Paris, France; INSERM U1149, Centre de Recherche sur L’Inflammation, University of Paris Cité, 46 Rue Henri Huchard, 75018 Paris, France; Department of Genetics, Bichat Hospital (Assistance Publique Hopitaux de Paris), Reference Laboratory for the Diagnosis of Birt Hogg Dube Syndrome, University of Paris Cité, 46 Rue Henri Huchard, 75018 Paris, France

Birt-Hogg-Dubé syndrome (BHD), an autosomal dominant disorder caused by pathogenic variants (PVs) in *FLCN*, presents variably with fibrofolliculomas, lung cysts, and renal cell carcinoma.^1^ Early diagnosis enables surveillance for renal cell carcinoma, thereby improving patient outcomes. Since 2009, our laboratory has performed molecular diagnoses of BHD, managing a large national patient cohort. Among 329 consecutive unrelated French BHD probands with *FLCN* PVs, we detected well-known variants c.1285dup and c.1285del in 54 patients (16.4%), a frequency consistent with previous reports.^1^, ^2^, ^3^ These hotspot variants arise from the intrinsic instability of the polycytosine tract. In contrast, we observed an unexpectedly high frequency of the c.1300G>A, p.(Glu434Lys), being present in 24 individuals (7.3%), all from whom were Caucasians originating from Normandy and Brittany. All other variants were much less frequent.

Globally, the c.1300G>A variant is exceedingly rare, having been reported in only five patients to date: one case in an American study^2^ and four cases in a French study,^3^ although the latter did not clarify whether these cases represented independent individuals or related members of the same family. The variant was absent from HGMD® Professional (2024.4), and detected in just 1 of 806,712 individuals in GnomAD v4.1.0 (allele frequency, AF = 6.2 × 10^−7^). The Leiden Open Variation Database (LOVD) classifies c.1300G>A as pathogenic, whereas ClinVar lists it as a variant of uncertain significance (VUS). *In silico* predictions suggest disruption of the exon 11 donor splice site (MaxEnt: −31.4%, Splice AI: 0.2).

To clarify the pathogenicity of c.1300G>A, we assessed its molecular impact. RNA-sequencing from peripheral blood (PAXgene®, Twist®; see Supplementary Material) demonstrated exon 11 skipping (Fig. 1A), with 28% aberrant transcripts (Nonsense-Mediated mRNA Decay, NMD). This finding provides strong evidence supporting reclassification of c.1300G>A as pathogenic, addressing its previous VUS status.

**Figure 1:**
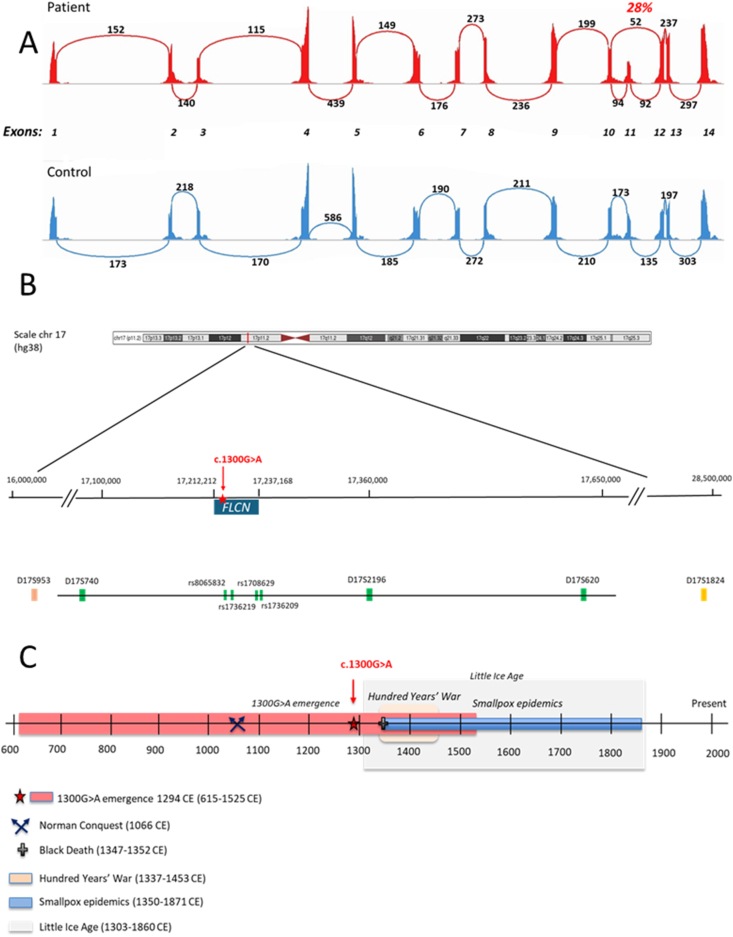
Pathogenicity, founder effect, and hypothetical origin of the c.1300G>A Variant. **(A)** RNA-seq showing alternative splicing with exon 11 skipping in heterozygous carrier, demonstrating the pathogenicity of c.1300G>A. Alternative transcript represented 28% of total transcripts (< 50%), indicating partial degradation via Nonsense-Mediated mRNA Decay (NMD). Gene model: exons as blocks, introns as lines. Coverage histograms: exon read density, junction arcs: splice junctions with read counts. **(B)** Shared haplotype (D17S740-12 to D17S620-10) in c.1300G>A carriers confirming a founder effect. Microsatellites (D17S) were genotyped by fragment analysis (ABI PRISM 3130xl, GeneMapper v5.0), and SNPs (rs) were obtained from sequencing data. **(C)** Timeline of c.1300G>A origin and historical events in Normandy and Brittany. The estimated origin (Austerlitz method, 29.25 generations, 25 years/generation) of *FLCN* c.1300G>A (∼1294 CE, 731 years ago; 95% CI: 615–1525 CE) is shown with Norman Conquest (1066 CE), the Black Death (1347–1352 CE), Hundred Years' War (1337–1453 CE), and smallpox epidemics (∼1350–1871 CE). The Little Ice Age (∼1303–1860 CE) provides climatic context. The timeline extends to 2025 CE to illustrate persistence of the variant.

To investigate the elevated frequency of this deleterious variant, we hypothesized a founder effect and tested this through haplotype analysis. Using microsatellite markers (ABI PRISM®, GeneMapper™) and SNP genotyping, we identified a shared 0.58 Mb haplotype (chr17:17,096,345–17,676,589; D17S740-12, rs8065832-A, rs1736219-A, rs1708629-A, rs1736209-G, D17S2196-8, D17S620-10), excluding outer markers D17S953 and D17S1824, in all 24 probands and their six c.1300G>A-carrying relatives (Fig. 1B). This haplotype was absent in nine non-carrier relatives and present in only one of 107 controls (100 from Normandy and Brittany), a Breton individual, supporting a founder mutation.

Using the Austerlitz method^4^ (Supplementary Material), we estimated an origin at around 1294 CE (29.25 generations; 95% CI: 615–1525 CE), accounting for *θ* = 0.7–1 cM/Mb and founder effect (*L*_0_ = 0.744 Mb, *N*_*e*_ ≈ 400–600). This aligns with medieval crises in Normandy and Brittany, such as the Black Death (1347–1352), the Hundred Years’ War (1337–1453), smallpox epidemics (1165–1871), and the Little Ice Age (1303–1860) (Fig. 1C). Given the persistence of this deleterious variant at such a high frequency, we explored potential selective mechanisms.

We modeled the variant's evolutionary trajectory from an initial frequency of *p*_0_ = 0.0000167 (*N*_e_ = 30,000) across 29 generations (Supplementary Material). Under a heterozygote advantage of *s* = 0.02, the Wright–Fisher model yielded *p*_29_ = 0.0000298 deterministically and *p*_29_ ≈ 0.000038 stochastically, incorporating a 50% population bottleneck around 1350 CE to reflect the Black Death's impact. Alternative scenarios, such as *s* = 0.01 (p ≈ 0.000028), *s* = 0.05 (*p* ≈ 0.00008), or a transient *s* = 0.05 during the plague (*p* ≈ 0.00002), consistently deviated from this range.

We propose that c.1300G>A offered a modest 2% fitness edge during medieval crises. *FLCN* regulates the mTOR and TGF-β pathways, which are linked to immunity and tissue repair.^5^ Heterozygotes might have benefited from enhanced pulmonary resilience, potentially via *FLCN's* regulation of mTOR, against plague or the Little Ice Age's harsh climates, or reduced smallpox scarring, improving survival in high-mortality settings. The 1350 CE bottleneck amplified this via drift, with later expansion preserving the haplotype. While drift alone is possible, *s* = 0.02's fit suggests a selective advantage.

The estimated c.1300G>A origin (∼1294 CE) postdates the Norman Conquest (1066 CE), yet the 95% CI (615–1525 CE) allows for an earlier emergence. However, its absence in the UK Biobank and British BHD cohorts supports a post-Conquest, France-specific event.

This observation echoes heterozygote advantages seen in some dominant disorders, where mutations may enhance adaptation to environmental pressures. It suggests that rare pathogenic *FLCN* variants might reflect transient adaptive benefits. While the proposed heterozygote advantage is an intriguing possibility, we acknowledge that genetic drift may modulate positive selection. Resolving this requires functional assays and larger genetic studies to explore how these variants alter cellular processes, their potential selective origins, and their evolutionary trajectories. To conclude, the pronounced enrichment of c.1300G>A in Normandy and Brittany reveals how historical selection intertwines with genomic epidemiology to define rare disease patterns.

## CRediT authorship contribution statement

**Agathe Hercent:** Writing – review & editing, Methodology, Investigation, Data curation. **Ibrahima Ba:** Writing – review & editing, Supervision, Investigation, Funding acquisition, Formal analysis, Data curation. **Dimitri Tchernitchko:** Writing – original draft, Supervision, Project administration, Methodology, Data curation, Conceptualization.

## Ethics declaration

All patients provided written informed consent for molecular analysis, in accordance with institutional ethical guidelines. The study was approved by the local ethics committee: Comité de Protection des Personnes (CPP) Ile de France 1, approval No: 0811760.

## Conflict of interests

The authors declare no conflict of interests.
