# Supplementary material for: FLCN c.1300G>A: Selective advantage in medieval France
Source: Genes Dis. 2025 Dec 30;13(6):102018. doi: 10.1016/j.gendis.2025.102018 (PMC13319673; doi:10.1016/j.gendis.2025.102018)
Supplement: Multimedia component 1 [file mmc1.docx]

**Supplementary Material**

1.1. Patient Cohort

All patients provided written informed consent for molecular analysis, in accordance with institutional ethical guidelines. The study was approved by the local ethics committee: Comité de Protection des Personnes (CPP) Ile de France 1; 0811760).

We analyzed clinical and molecular data from patients referred to the Department of Genetics at Bichat University Hospital (Paris, France) for *FLCN* gene screening between January 1, 2009, and March 31, 2025. Referrals were primarily for suspected Birt-Hogg-Dubé syndrome (BHD) based on clinical features such as fibrofolliculomas, lung cysts, renal cell carcinoma, or family history. For each patient, prescribing clinicians completed a standardized BHD clinical form documenting clinical findings, paraclinical results (e.g., imaging, histopathology), and family history. Data were extracted from our laboratory’s secure database, with additional clinical and paraclinical information retrieved from anonymized medical records. Data were anonymized and stored in a password-protected digital database compliant with GDPR regulations.

1.2. DNA Extraction and Molecular Screening

Genomic DNA was extracted from peripheral whole blood collected in EDTA tubes using the Chemagic Star DNA Extractor (Hamilton Robotics). From 2009 to 2014, *FLCN* molecular screening was performed using Sanger sequencing and multiplex ligation-dependent probe amplification (MLPA). Sanger sequencing targeted all *FLCN* exons and flanking intronic regions (±20 bp). DNA was amplified using custom-designed primers (sequences available upon request). Purified PCR products were sequenced bidirectionally using the BigDye Terminator v3.1 Cycle Sequencing Kit on an ABI PRISM 3130xl Genetic Analyzer (Applied Biosystems). Sequences were analyzed with SeqScape v2.7 software, aligned to the *FLCN* reference sequence (NM_144997.7). Copy number variations (CNVs) were detected using the SALSA MLPA P256-B2 *FLCN* Probemix (MRC Holland), with data analyzed via Coffalyser.Net software.

From 2015 onward, next-generation sequencing (NGS) replaced Sanger sequencing. Between 2015 and 2021, a custom AmpliSeq panel (Thermo Fisher Scientific) was used, covering all *FLCN* exons and flanking intronic regions (±20 bp). From 2021 to 2025, a custom capture-based panel (TWIST Bioscience) was employed, sequenced on the NextSeq 550 System (Illumina). Both NGS panels achieved a minimum coverage depth of 30X per base, with >98% of targeted regions covered at ≥100X. Sequencing data were processed using QIAGEN CLC Genomics Workbench (v22.0). Variant calling was performed with a minimum allele frequency threshold of 5%, followed by annotation against databases including ClinVar, LOVD, and HGMD Professional (2024.4). Variants were classified according to the American College of Medical Genetics and Genomics (ACMG) and Association for Molecular Pathology (AMP) guidelines.^1^ All identified variants were confirmed by Sanger sequencing.

1.3. Haplotype Analysis with Microsatellite Markers and SNP Genotyping

To investigate the founder effect of the c.1300G>A mutation in the *FLCN* gene, we analyzed five microsatellite markers (D17S740 (CA)n, D17S2196 (GATA)n, D17S620 (TG)n, D17S953 (CA)n, D17S1824 (AATG)n) in 24 probands, six relatives carrying c.1300G>A, two related non-carriers, and 20 unrelated controls from Normandy and Brittany. The markers were selected based on their proximity to the FLCN locus (chr17:17,157,408-17,182,227, GRCh38) and high heterozygosity, as reported in the UniSTS database. DNA was amplified using fluorescently labeled primers (6-FAM dyes) designed for each microsatellite locus (primer sequences and PCR conditions available upon request). Fragment analysis was conducted on an ABI PRISM 3130xl Genetic Analyzer using a 36 cm capillary array and POP-7 polymer. Allele sizes were determined with GeneMapper v5.0 software (Applied Biosystems), using a peak height threshold of 200 RFU for reliable calling. Alleles were assigned based on fragment sizes corresponding to the number of repeat units, calibrated against a LIZ-500 size standard, and a shared haplotype was identified in all c.1300G>A carriers, defined by alleles D17S740-12 (12 CA repeats), D17S2196-8 (8 GATA repeats), and D17S620-10 (10 TG repeats), as well as intragenic SNPs rs8065832-A, rs1736219-A, rs1708629-A, and rs1736209-G, spanning a 0.58 Mb region (chr17:17,096,345-17,676,589, GRCh38). SNP genotyping was performed using sequencing data generated from Sanger sequencing (2009–2014) and next-generation sequencing (NGS, 2015–2025) of *FLCN*, as described in section 1.2. Variants were identified and validated using SeqScape v2.7 for Sanger data and QIAGEN CLC Genomics Workbench v22.0 for NGS data. Flanking markers D17S953 and D17S1824 showed recombination, defining haplotype boundaries. The haplotype was absent in non-carriers relatives and controls, supporting a founder effect.

1. RNA Sequencing Analysis for c.1300G>A Pathogenicity

To assess the molecular impact of the c.1300G>A mutation, we performed RNA sequencing (RNA-seq) to evaluate its effect on splicing, focusing on exon 11 skipping as predicted by in silico tools.

Total RNA was extracted from peripheral blood samples collected in PAXgene Blood RNA Tubes (PreAnalytiX, Qiagen/BD) from heterozygous c.1300G>A carriers and non-carrier controls. RNA extraction followed the PAXgene Blood RNA Kit (Qiagen, cat. no. 762174) protocol.

RNA-seq libraries were prepared using the TWIST Bioscience RNA Library Prep Kit. Sequencing was performed on an Illumina NextSeq 550 System using a High Output Kit v2.5

FASTQ files were quality-checked with FastQC (v0.11.9). Reads were aligned to GRCh38 using STAR alignment: two-pass mode, --outSAMtype BAM SortedByCoordinate. Gene annotation used GENCODE v38.

Splicing was visualized using Sashimi Plots in IGV (v2.12.3). Plots were generated with ggsashimi (v1.1.0, minimum 5 reads), showing read density and junction arcs. For carriers, elevated exon 10–exon 12 junction reads confirmed exon 11 skipping.

1. Estimation of Mutation Origin Using the Austerlitz Method

3.1. Haplotype Analysis

Haplotype analysis was performed as described in 1.3, confirming a 0.58 Mb shared haplotype (chr17:17,096,345-17,676,589, GRCh38) in all c.1300G>A carriers. Recombination events at D17S953 and D17S1824 delineated the haplotype boundaries.

3.2. Recombination Rate and Haplotype Length

The recombination rate (θ) for chromosome 17p11.2 was estimated at 0.85 cM/Mb (range: 0.7–1 cM/Mb).^2^ The shared haplotype length (Lₜ = 0.58 Mb) was consistent across all 24 carriers from Normandy and Brittany. Recombination boundaries were refined to chr17:17,096,345-17,676,589 (GRCh38), we calculated:

$\theta t=Lt\times r=0.58 \mathrm{Mb}\times0.85 cM/Mb=0.00493 \mathrm{Morgans}$

To account for the founder effect, the initial haplotype length (L₀) was adjusted to 0.744 Mb (vs. 0.9 Mb in panmictic populations), reflecting genetic drift in a small ancestral population (Nₑ ≈ 400–600).

3.3. Age Estimation

Using the Austerlitz method ^3^:

$g=\frac{\ln\left( L0/Lt \right)}{\theta\mathrm{Mb}}$ = $\frac{\ln\left( 0.744/0.58 \right)}{0.0085}\approx29.25 \mathrm{generations}$

Assuming 25 years per generation, the mutation arose:

Age=29.25×25=731 years (∼1294 CE; 95% CI: 615–1525 CE),

accounting for: recombination rate variability (θ = 0.7–1 cM/Mb), ancestral haplotype uncertainty (L₀ = 0.7–0.8 Mb), generation length (25–30 years).

1. Evolutionary Modeling of c.1300G>A Frequency

To investigate the persistence of the c.1300G>A mutation despite its deleterious effects, we modeled its allele frequency evolution over 29.25 generations (~731 years, ~1294 CE to 2025 CE) using deterministic and stochastic Wright-Fisher models, assuming selection as the primary evolutionary force with genetic drift and a historical population bottleneck.^4^

4.1. Initial Parameters

The initial allele frequency was set to $p\_0 = 1 / (2 \times30,000)$= 0.0000167, where N_e = 30,000 is the effective population size in Normandy and Brittany before the Black Death (~1350 CE). Modeling spanned 29.25 generations, consistent with the mutation’s estimated origin (~1294 CE, section 3.3).

4.2. Deterministic Wright-Fisher Model

We applied the deterministic Wright-Fisher model, assuming an infinite population and non-overlapping generations.^4^ Fitness values were defined as w_AA = 1 (wild-type homozygotes), w_Aa = 1.02 (heterozygotes), and w_aa = 0.9 (mutant homozygotes), reflecting a 2% heterozygote advantage (s = 0.02). The allele frequency at generation t+1 was calculated as: $p\_(t+1) =$ $[p\_t^2 \times w\_AA + p\_t \times(1 - p\_t) \times w\_Aa] / w̄$,

where w̄ = $p\_t^2 \times w\_AA + 2 \times p\_t \times(1 - p\_t) \times w\_Aa + (1 - p\_t)^2 \times w\_aa$is the mean population fitness. Iterative application over 29.25 generations yielded p_29 ≈ 0.0000298, indicating a modest increase in allele frequency under s = 0.02, selected for its fit to the observed frequency and historical context (section 4.4).

4.3. Stochastic Model with Bottleneck

To account for genetic drift and the Black Death’s impact (~1350 CE), we implemented a stochastic Wright-Fisher model with a 50% population bottleneck reducing N_e from 30,000 to 15,000 at generation ~2 (corresponding to ~1350 CE, ~56 years after mutation origin).^4,5^

The number of mutant alleles at generation t+1 was modeled as

$N\_a(t+1) \sim Binomial(2 \times N\_e(t)$, $p\_t \times w\_Aa / w̄)$, with w̄ as defined in 4.2.

Monte Carlo simulations (10,000 replicates) over 29.25 generations yielded p_29 ≈ 0.000038 (95% CI: 0.00001–0.00009), reflecting increased frequency due to drift during the bottleneck.

4.4. Historical Context and Selective Hypothesis

The mutation’s estimated origin (~1294 CE, section 3.3) precedes medieval crises in Normandy and Brittany, including the Black Death (1347–1352 CE), Hundred Years’ War (1337–1453 CE), smallpox epidemics (~1350–1871 CE), and Little Ice Age (~1303–1860 CE). We hypothesize that a 2% heterozygote advantage (s = 0.02) enhanced survival in heterozygotes, potentially via *FLCN*-mediated mTOR and TGF-β pathways regulating immunity and tissue repair.^6^ Enhanced pulmonary resilience or reduced smallpox scarring may have improved survival in high-mortality settings. The bottleneck amplified this advantage through drift, with post-crisis population expansion preserving the haplotype.

4.5. Validation and Sensitivity Analysis

Sensitivity analyses tested alternative scenarios: s = 0.01 (p_29 ≈ 0.000028), s = 0.05 (p_29 ≈ 0.00008), and a transient s = 0.05 during the bottleneck (p_29 ≈ 0.00002). The s = 0.02 model (p_29: 0.000025–0.000045) best fit the observed frequency, supporting a modest selective advantage robust to variations in N_e (10,000–20,000) and bottleneck timing (±2 generations). These results suggest that while drift contributed, selection likely played a role in the mutation’s persistence.

**References (Supplementary Material)**

1. Richards S, Aziz N, Bale S, et al. Standards and guidelines for the interpretation of sequence variants: a joint consensus recommendation of the American College of Medical Genetics and Genomics and the Association for Molecular Pathology. Genet Med. 2015;17(5):405-424. doi:10.1038/gim.2015.30
2. Kong A, Thorleifsson G, Gudbjartsson DF, et al. Fine-scale recombination rate differences between sexes, populations and individuals. Nature. 2010;467(7319):1099-1103. doi:10.1038/nature09525
3. Austerlitz F, Kalaydjieva L, Heyer E. Detecting population growth, selection and inherited fertility from haplotypic data in humans. Genetics. 2003;165(3):1579-1586. doi:10.1093/genetics/165.3.1579
4. Hartl DL, Clark AG. Principles of Population Genetics. 4th ed. Sinauer Associates; 2007.
5. Benedictow OJ. The Black Death, 1346–1353: The Complete History. Boydell Press; 2004.
6. Liu GY, Sabatini DM. mTOR at the nexus of nutrition, growth, ageing and disease. Nat Rev Mol Cell Biol. 2020;21(4):183-203. doi:10.1038/s41580-019-0199-y
